# No Association of *Trichomonas vaginalis* Seropositivity with Advanced Prostate Cancer Risk in the Multiethnic Cohort: A Nested Case-Control Study

**DOI:** 10.3390/cancers15215194

**Published:** 2023-10-28

**Authors:** Michelle Nagata, Anne Tome, Kami White, Lynne R. Wilkens, Song-Yi Park, Loïc Le Marchand, Christopher Haiman, Brenda Y. Hernandez

**Affiliations:** 1 Population Sciences in the Pacific Program, University of Hawaii Cancer Center, 701 Ilalo Street, Honolulu, HI 96813, USA; atome@cc.hawaii.edu (A.T.); kwhite@cc.hawaii.edu (K.W.); lynne@cc.hawaii.edu (L.R.W.); spark@cc.hawaii.edu (S.-Y.P.); loic@cc.hawaii.edu (L.L.M.); brenda@cc.hawaii.edu (B.Y.H.); 2 Center for Genetic Epidemiology, Keck School of Medicine, University of Southern California, 1975 Zonal Ave., Los Angeles, CA 90033, USA; christopher.haiman@med.usc.edu

**Keywords:** prostate, prostate cancer, advanced prostate cancer, *Trichomonas vaginalis*, trichomoniasis

## Abstract

**Simple Summary:**

Prostate cancer is one of the most common malignancies in the United States and worldwide, with geographic variability in incidence and mortality. Despite increasing incidence of distant-stage prostate cancer, the cause is poorly understood. There is inconsistent serologic evidence that *Trichomonas vaginalis*, a sexually transmitted protozoan, may play a role in prostate cancer development. The aim of our study was to assess the relationship between *T. vaginalis* seropositivity and advanced prostate cancer risk in a nested case–control study within the Multiethnic Cohort in Hawaii and California using blood samples collected prior to cancer diagnoses. Understanding the relationship between *T. vaginalis* seropositivity and prostate cancer risk in this diverse cohort will inform treatment and prevention efforts and address disparities in incidence and mortality.

**Abstract:**

The potential involvement of a sexually transmitted agent has been suggested to contribute to the high number of prostate cancers in the United States and worldwide. We investigated the relationship of *Trichomonas vaginalis* seropositivity with prostate cancer risk in a nested case–control study within the Multiethnic Cohort in Hawaii and California using blood samples collected prior to cancer diagnoses. Incident cases of advanced prostate cancer (intermediate- to high-grade based on Gleason score ≥ 7 and/or disease spread outside the prostate) were matched to controls by age, ethnicity, and the date of blood collection. *T. vaginalis* serostatus was measured using an ELISA detecting IgG antibodies against a recombinant *T. vaginalis* α-actinin protein. Seropositivity to *T. vaginalis* was observed in 35 of 470 (7.4%) cases and 26 of 470 (5.5%) controls (unadjusted OR = 1.47, 95% CI 0.82–2.64; adjusted OR = 1.31, 95% CI 0.67–2.53). The association was similarly not significant when cases were confined to extraprostatic tumors having regional or distant spread (*n* = 121) regardless of grade (unadjusted OR = 1.37, 95% CI 0.63–3.01; adjusted OR = 1.20, 95% CI 0.46–3.11). The association of *T. vaginalis* with prostate cancer risk did not vary by aspirin use. Our findings do not support a role for *T. vaginalis* in the etiology of advanced prostate cancer.

## 1. Introduction

Although prostate cancer is one of the most common malignancies in the United States and worldwide [1] and the incidence of distant stage prostate cancer is growing [2], its etiology is poorly understood. Age, race, family history, and genetic predisposition are the only well-established risk factors [1,3], with variability in incidence and mortality by geographic location [3]. The potential involvement of a sexually transmitted agent in prostate cancer development is suggested by the evidence linking sexual activity to prostate cancer risk, including multiple partners [4] and an early age of initial sexual activity [5]. A number of case–control studies have observed an association of prostate cancer with history of sexually transmitted infections (STI) [6,7,8] including gonorrhea [4,9,10] and syphilis [9,10]. A possible infectious etiology of prostate cancer is also supported by inconsistent evidence linking prostatitis with prostate cancer risk [4,9,11]. It has been suggested that inflammation of the prostate may be caused by infectious agents accessing the prostate via ascension from the urethra [12]. A number of STIs have been detected in the prostatic secretions and urine of men with acute and chronic prostatitis, including *Chlamydia trachomatis* and *Trichomonas vaginalis* [13,14,15]. Serologic evidence linking STIs with prostate cancer risk has been largely null for human papillomavirus (HPV), human herpesvirus type 8, and *Chlamydia trachomatis* [16,17,18].

*Trichomonas vaginalis* is a sexually transmitted protozoan, which usually establishes an asymptomatic infection in men [19]. In 2018, there were an estimated 3.3 million new infections in U.S. men aged 18–59 [19]. In addition to its potential link to chronic prostatitis [14], trichomonads have been detected in the prostate including benign prostatic hyperplasia [20]. There is inconsistent serologic evidence that *T. vaginalis* may play a role in prostate cancer development [21]. In a large, nested case–control study (*n* = 691 case–control pairs), *T. vaginalis* seropositivity was associated with prostate cancer after adjustment for history of other STI and history of clinical prostatitis (odds ratio [OR] = 1.43, 95% confidence interval [CI] 1.00–2.03) [22]. This association was not significant when only high-grade prostate tumors classified by Gleason score ≥ 7 (*n* = 243 case–control pairs) were assessed (OR = 1.76, 95% CI 0.97–3.18). The magnitude of the association between *T. vaginalis* seropositivity and prostate cancer incidence was greatest among men with infrequent lifetime aspirin use (*n* = 186 cases, *n* = 163 controls) (OR = 2.05, 95% CI 1.05–4.02), suggesting inflammation as a mechanism by which *T. vaginalis* might induce prostate cancer [22]. *T. vaginalis* has been reported to stimulate production of inflammatory cytokines such as IL-6 in prostate epithelial cells [23]. Other proposed mechanisms include the elevated secretion of polyamines and upregulation of anti-apoptotic oncogenes that affect cell proliferation [24,25]. Later studies corroborated a positive association between *T. vaginalis* seropositivity and prostate cancer risk [25,26] as well as between *T. vaginalis* seropositivity and higher prostate-specific antigen (PSA) levels and tumor stage [25].

In contrast to these findings, several case–control studies found no association between *T. vaginalis* seropositivity and prostate cancer [27,28,29,30,31]. Most reported null associations for advanced prostate cancers [29,31]. Another study (*n* = 105 cases, *n* = 673 controls) found increased risk only for cases of extraprostatic prostate cancer, in which local tumor cells extended beyond the prostate (OR = 2.17, 95% CI 1.08–4.37), and of cancer that would progress to bone metastases or prostate-cancer specific death [28]. We investigated the relationship between *T. vaginalis* seropositivity and advanced prostate cancer risk in a nested case–control study within the Multiethnic Cohort (MEC) in Hawaii and California using blood samples collected prior to cancer diagnoses.

## 2. Materials and Methods

### 2.1. Study Cohort

We conducted a nested case–control study within the MEC to test the hypothesis that seropositivity for *T. vaginalis* increased the risk of prostate cancer. The MEC is composed of more than 215,000 adults aged 45–75 living in Hawaii and California [32]. Participants were recruited in 1993–1996, primarily from five racial and ethnic populations: African American, Japanese American, Latino, Native Hawaiian, and White. At study enrollment, all participants completed a mailed questionnaire detailing demographic, medical, and dietary history information. A prospective biospecimen repository was developed during the follow-up period, largely between 2001 and 2006, consisting of more than 70,000 participants who provided blood and/or urine specimens as well as updated information on a few items from the baseline questionnaire. The study was approved by the institutional review boards overseeing research on human subjects at the University of Hawaii and the University of Southern California. All biorepository participants provided written informed consent. Details of the design and development of the MEC have been previously described [32].

### 2.2. Nested Case-Control Study Population

Incident advanced prostate cancer cases were identified through linkage to the cancer registries covering the states of Hawaii and California, which are part of the Surveillance, Epidemiology and End Results (SEER) Program of the National Cancer Institute. Advanced prostate cancer cases were defined as individuals with histologically confirmed, invasive prostate cancer tumors spread outside the prostate (i.e., regional or metastatic disease) and/or with intermediate- to high-grade prostate tumors based on Gleason score ≥ 7 who were diagnosed after blood collection up to the 2006 cancer registry linkages. For each case, one control was randomly selected from a biorepository pool of males who were alive and free of prostate cancer at the time of the case’s diagnosis and who matched the case on age at cohort entry (±5 years), ethnicity (White, African American, Native Hawaiian, Japanese American, Latino), and date of blood draw (±2 years). A total of 470 advanced prostate cancer cases and 470 matched controls were included in the present study.

### 2.3. Serologic Testing

*T. vaginalis* antibody was evaluated in the laboratory of Dr. John Alderete of Washington State University using an ELISA detecting IgG against a highly immunogenic recombinant α-actinin protein of *T. vaginalis* [22]. Frozen serum samples were shipped to the Alderete laboratory, which was blinded to the case–control status of specimens. Absorbance values of the antibody were measured at A405 using an ELISA reader (Bio-TEK Instruments, Inc., Winooski, VT, USA). All prostate cancer case and control samples were tested in duplicate and absorbance scores for each sample were based on the mean score of the duplicate samples. A panel of duplicate positive and negative controls were included on each plate. Cut-off values were determined by the ratio of the mean absorbance of positive controls to that of negative controls. Samples with absorbance scores above the cut-off were considered to be positive.

### 2.4. Statistical Analysis

The statistical analysis focused on the relationship of *T. vaginalis* with advanced prostate cancer risk in cases and controls. All analyses were performed using SAS statistical software version 9.4 (SAS Institute, Cary, NC, USA). Analysis of demographic and medical differences between cases and controls was performed using chi-squared tests for categorical variables and *t*-tests for continuous variables. Conditional logistic regression of prostate cancer with matched case–control sets as strata was used to estimate ORs and 95% CIs. Variables observed to differ between cases and controls as well as other potentially confounding variables were included as covariates in the multivariate models. Logistic regression analysis of extraprostatic tumors compared cases with regional and distant cancer to all controls. Two-sided *p*-values ≤ 0.05 were considered significant for all statistical tests.

## 3. Results

Case (*n* = 470) and control (*n* = 470) groups each included 205 (43.6%) Japanese Americans, 129 (27.5%) Whites, 55 (11.7%) African Americans, 42 (8.9%) Native Hawaiians, and 39 (8.3%) Latinos (Table 1). Mean (SD) age at diagnosis was 72.7 (7.77) years, and mean (SD) age at cohort entry was 61.0 (7.8) years for cases and 62.3 (7.6) years for controls (*p* = 0.011). Mean (SD) age at blood draw was 70.0 (7.67) years among cases and 71.3 (7.46) years among controls (*p* = 0.008).

Advanced prostate tumors among cases included 349 (74%) localized tumors, 78 (17%) tumors with regional spread, and 43 (9.2%) with distant metastatic disease. The majority of tumors were classified as intermediate- or high-grade with a Gleason score ≥ 7 (440/470, 94%), 19 (4.0%) were low-grade with a Gleason score ≥ 6, 4 (0.85%) were undifferentiated, and 7 (1.5%) were of unknown grade. Compared to controls, prostate cancer cases included a higher proportion of college graduates (43.1% of cases vs. 35.7% of controls, *p* = 0.08) and those with a family history (father and/or brother) of prostate cancer (11.2% of cases vs. 6.6% of controls, *p* = 0.016) as well as lower proportions of current smokers (10.5% of cases vs. 22.8% of controls, *p* < 0.001), those with a history of heart attack (6.0% of cases vs. 11.1% of controls, *p* = 0.005), and current aspirin users (23.2% of cases vs. 30.0% of controls, *p* = 0.023). Cases and controls were not significantly different with respect to other medical and demographic factors including a history of enlarged prostate, marital status, and number of children.

Seropositivity to *T. vaginalis* was observed in 35 of 470 (7.4%) cases and 26 of 470 (5.5%) controls (unadjusted OR = 1.47, 95% CI 0.82–2.64) (Table 2). The association between *T. vaginalis* seropositivity and prostate cancer cases remained non-significant when adjustment was made for years of education, history of smoking, history of heart attack, and family history of prostate cancer (adjusted OR = 1.31, 95% CI 0.67–2.53). The association was similar when cases were confined to those with extraprostatic extension (*n* = 121), regardless of grade (unadjusted OR = 1.37, 95% CI 0.63–3.01; adjusted OR = 1.20, 95% CI 0.46–3.11). The association between *T. vaginalis* and prostate cancer risk did not vary by aspirin use. The association, however, was in a more positive albeit non-significant direction among those who did not use aspirin (adjusted OR = 1.19, 95% CI 0.31–4.60) compared to ever users (adjusted OR = 1.06, 95% CI 0.24–4.74) and among non-current aspirin users (adjusted OR = 1.30, 95% CI 0.40–4.23) compared to current users (adjusted OR = 0.87, 95% CI 0.12–6.24).

## 4. Discussion

Our findings from this nested case–control study do not support a role for *T. vaginalis* in the etiology of advanced prostate cancer. Previous studies in other cohorts, similarly, reported no association between *T. vaginalis* infection and prostate cancer incidence [27,29,30,31]. Furthermore, *T. vaginalis* seropositivity has not been observed to increase prostate cancer-specific or all-cause mortality among prostate cancer patients [33]. While a few reports have suggested a positive association between *T. vaginalis* infection and prostate cancer risk [22,25,26], differences in participant demographic characteristics and methods for detecting *T. vaginalis* infection and diagnosing prostate cancer may have contributed to this discrepancy [25]. Seropositivity was lower among both cases (7.4%) and controls (5.5%) in the MEC compared to other cohorts, including the Health Professionals Follow-up Study (12.6% and 9.4%) [22] and Physicians’ Health Study (24.5% and 21.4%) [28].

Differences in inclusion criteria may account for discrepancies. Our study evaluated clinically relevant advanced prostate cancers, defined by regional or metastatic spread and/or intermediate- to high-grade tumors based on a Gleason score ≥ 7. We also assessed cases with extraprostatic spread because a previous study by Stark et al. found a positive association between *T. vaginalis* seropositivity and prostate cancer risk only for extraprostatic cases and metastatic or fatal prostate cancers [28]. Another study demonstrated a null association for advanced cases with regional or metastatic spread and/or Gleason score ≥ 8 [31]. Studies reporting an overall positive association between *T. vaginalis* seropositivity and prostate cancer risk noted too few advanced stage cases (regional or metastatic spread) to assess for an association [22] or did not define cancer stages or grades [26].

Previous case–control studies have evaluated *T. vaginalis* infection in predominantly White men [22,27,29], and limited studies have included a sizeable number of Black men [30,31]. These studies reported no association between *T. vaginalis* infection and prostate cancer risk for African American and White men [30,31]. Our study within the MEC was unique in that we included African Americans, Japanese Americans, Latinos, Native Hawaiians, and Whites. Previous studies on the MEC found increased prostate cancer risk for African Americans and Latinos compared to Whites [34,35]. National trends corroborate higher prostate cancer incidence and mortality rates for Blacks compared to Whites [36]. A higher prevalence of *T. vaginalis* infection has also been reported for Blacks compared to other races [37]. Additionally, while most previous study populations had higher educational levels and socioeconomic statuses [31] and/or were limited to participants who were physicians and health professionals [22,28], the MEC encompasses a range of both educational and socioeconomic levels [32].

Our study was limited by marginal power for detecting significant associations between *T. vaginalis* infection and prostate cancer incidence found in previous studies. Our analysis of advanced prostate cancer cases in the MEC had 67% power, though we achieved 86% power when evaluating those with infrequent aspirin use, who had the highest prostate cancer incidence in the Health Professionals Follow-up Study. The Health Professionals Follow-up Study, similarly, found no association between *T. vaginalis* seropositivity and the incidence of prostate cancer, with a Gleason score ≥ 7 [22]. Our study achieved 76% power when evaluating extraprostatic cases. The Physicians’ Health Study reported increased *T. vaginalis* infection among extraprostatic cases only [28].

In our study, the detection of *T. vaginalis* infection was limited to serological analysis using ELISA. Although ELISA cannot discriminate between recent and remote infection [38], this is the same methodology used in previous case–control studies investigating *T. vaginalis* infection and prostate cancer risk [22,25,27,28,29,30,31]. While serological methods for diagnosing *T. vaginalis* are rarely used clinically, high sensitivity and specificity have been reported for monoclonal antibody-based ELISA in *T. vaginalis* detection [39]. Data on the history of *T. vaginalis* infection should be collected to inform chronic trichomoniasis for future investigation.

In addition to evaluating *T. vaginalis* seropositivity, studies have assessed the effect of IL-6, a proinflammatory cytokine produced in response to *T. vaginalis* infection, on prostate cancer development [23]. The assessment of mouse models in vivo and human cell lines in vitro showed that IL-6 produced in prostate epithelial cells exposed to *T. vaginalis* induced the polarization of M2 macrophages [23]. M2 macrophages are the main tumor-associated macrophages found to promote the survival, proliferation, and metastasis of cancer cells through their critical roles in chronic inflammation, tissue repair, and angiogenesis [23]. It should be noted that although IL-6 has been targeted for its proliferative and antiapoptotic effects, variable IL-6 responses have been observed in prostate cancer [40]. A previous study demonstrating an association between *T. vaginalis* seropositivity and prostate cancer investigated the potential role of aspirin in reducing proliferative inflammatory lesions that serve as markers of a cellular environment conducive to prostate cancer development [22]. A positive association between *T. vaginalis* infection and prostate cancer was observed for men with infrequent lifetime aspirin use (0–19% of the time), and there was no association among regular users (80–100% of lifetime) [22]. In our study, although the association was stronger for non-users compared to users and among non-current users compared to current users, the association was not significant.

A few prostate cancer risk factors, including race/ethnicity, weight gain, family history, education, cigarette smoking, and diabetes, have been identified in the present cohort, and were found to be generally consistent with other studies. In addition to an increased prostate cancer risk for African Americans and Latinos [34,35], weight gain was also associated with prostate cancer risk in the MEC, although the relationship varied by race/ethnicity [41]. We found that, consistent with other studies [42,43], prostate cancer risk was increased for those with a positive family history and higher education level, which may reflect a higher screening rate [44]. Cigarette smoking, which was previously found to be related to lack of PSA screening in this cohort, was inversely associated with prostate cancer risk [45]. This inverse relationship is corroborated by a meta-analysis reporting an inverse association between current cigarette smoking and prostate cancer incidence in recent years [42]. Similar to other studies [42] diabetes was also associated with a decreased risk of prostate cancer [34,35,45].

Our findings, as well as potential risk factors identified in the MEC, are consistent with most previous reports. Although our study found no association between *T. vaginalis* seropositivity and advanced and/or extraprostatic prostate cancer risk, the increasing incidence of distant stage prostate cancer [2] and variability in prostate cancer incidence and mortality by geographic location [3] underline the need to further investigate its etiology in other cohorts. Additional efforts should assess inflammatory pathway responses to *T. vaginalis* infection that may contribute to prostate cancer progression.

## 5. Conclusions

Seropositivity to *T. vaginalis* was not associated with advanced prostate cancer risk within our ethnically diverse cohort of advanced prostate cancer cases and matched controls. The association remained insignificant when cases were confined to extraprostatic tumors having regional or distant spread, regardless of grade, and did not vary by aspirin use. Our findings do not support a role for *T. vaginalis* in the etiology of prostate cancer and contribute to current knowledge surrounding a possible infectious etiology of prostate cancer.

## Figures and Tables

**Table 1:** Demographic and medical characteristics of prostate cancer cases and matched controls within the Multiethnic Cohort.

Demographic/Medical Characteristic ^a^	No. (%)		*p*-Value ^b^
	Cases	Controls	
Age at cohort entry, mean (SD) years	61.0 (7.8)	62.3 (7.6)	0.011
Age at blood collection, mean (SD) years	70.0 (7.7)	71.3 (7.5)	0.008
Ethnicity			1.0
Japanese American	205 (43.6)	205 (43.6)	
White	129 (27.5)	129 (27.5)	
African American	55 (11.7)	55 (11.7)	
Native Hawaiian	42 (8.9)	42 (8.9)	
Latino	39 (8.3)	39 (8.3)	
Clinical or pathological stage		-	-
Localized	349 (74)		
Regional	78 (17)		
Distant	43 (9.2)		
Cancer grade		-	-
Intermediate or high-grade (Gleason score ≥ 7)	440 (94)		
Low-grade (Gleason score ≤ 6)	19 (4.0)		
Undifferentiated	4 (0.85)		
Education			0.08
≤8th grade	13 (2.8)	21 (4.5)	
9th–12th grades	127 (27.3)	146 (31.4)	
Vocational school/some college	125 (26.8)	132 (28.4)	
Graduated college	201 (43.1)	166 (35.7)	
Family history of prostate cancer in father or brother(s)			0.016
No	388 (88.8)	412 (93.4)	
Yes	49 (11.2)	29 (6.6)	
History of smoking			<0.001
Never	158 (34.0)	120 (25.9)	
Past smoker	258 (55.5)	238 (51.3)	
Current smoker	49 (10.5)	106 (22.8)	
History of heart attack			0.005
No	442 (94.0)	418 (88.9)	
Yes	28 (6.0)	52 (11.1)	
History of aspirin use			0.023
No	281 (60.8)	270 (58.3)	
Yes, but not currently	74 (16.0)	54 (11.7)	
Yes, currently	107 (23.2)	139 (30.0)	
History of enlarged prostate			0.92
No	394 (83.8)	395 (84.0)	
Yes	76 (16.2)	75 (16.0)	
Marital status			0.83
Married	387 (82.7)	380 (81.0)	
Separated or divorced	44 (9.4)	47 (10.0)	
Widowed	14 (3.0)	13 (2.8)	
Never married	23 (4.9)	29 (6.2)	
Number of children			0.72
None	62 (13.4)	72 (15.7)	
One	60 (12.9)	57 (12.5)	
Two	133 (28.7)	121 (26.4)	
Three or more	209 (45.4)	208 (45.4)	

^(a)^ Missing or unknown values were excluded. ^(b)^ Chi-squared tests and *t*-tests were used to compare categorical and continuous variables, respectively, for cases and controls matched by age at cohort entry (±5 years), ethnicity, and date of blood collection (±2 years).

**Table 2:** *Trichomonas vaginalis* serologic status and advanced prostate cancer risk in the Multiethnic Cohort.

*T. vaginalis* IgG		Cases (n = 470)		Controls ^a^ (n = 470)		Unadjusted OR	95% CI	Adjusted OR ^b^	95% CI
		n	%	n	%				
All cases and all controls ^c^	Seronegative	435	92.6	444	94.5	1.00	Reference	1.00	Reference
	Seropositive	35	7.4	26	5.5	1.47	0.82–2.64	1.31	0.67–2.53
Extraprostatic cases and all controls ^d^	Seronegative	112	92.6	114	94.2	1.00	Reference	1.00	Reference
	Seropositive	9	7.4	7	5.8	1.37	0.63–3.01	1.20	0.46–3.11

^(a)^ Matched to cases on age at cohort entry (±5 years), ethnicity (White, African American, Native Hawaiian, Japanese American, and Latino), and date of blood draw (±2 years). ^(b)^ Adjusted for years of education, history of smoking, history of heart attack, and family history of prostate cancer. ^(c)^ Based on conditional logistic regression. ^(d)^ Based on logistic regression; excluded cases with localized tumors.

## Data Availability

The data presented in this study are available on request from the corresponding author. The data are not publicly available due to privacy restrictions.
